# Investigation on the photoconductive behaviors of an individual AlN nanowire under different excited lights

**DOI:** 10.1186/1556-276X-7-454

**Published:** 2012-08-11

**Authors:** Fei Liu, Lifang Li, Tongyi Guo, Haibo Gan, Xiaoshu Mo, Jun Chen, Shaozhi Deng, Ningsheng Xu

**Affiliations:** 1 State Key Laboratory of Optoelectronic Materials and Technologies, Guangdong Province Key Laboratory of Display Material and Technology, Sun Yat-sen University, Guangzhou, 510275, People’s Republic of China; 2 School of Physics and Engineering, Sun Yat-sen University, Guangzhou, 510275, People’s Republic of China

**Keywords:** UAN, Photoconductive behaviors, Different wavelengths of illumination

## Abstract

Ultra-long AlN nanowire arrays are prepared by chemical vapor deposition, and the photoconductive performances of individual nanowires are investigated in our self-built measurement system. Individual ultra-long AlN nanowire (UAN) exhibits a clear photoconductive effect under different excited lights. We attribute the positive photocurrent response of individual UAN to the dominant molecular sensitization effect. It is found that they have a much faster response speed (a rise and decay time of about 1 ms), higher photocurrent response (2.7×10^6^), and more reproductive working performance (the photocurrent fluctuation is lower than 2%) in the air environment. Their better photoconductive performances are comparable to many nanostructures, which are suggested to be a candidate for building promising photosensitive nanodevices in the future.

## Background

Nanomaterials are very suitable for photosensitive device applications because their large surface-to-volume ratio and nanometer size are more sensitive to light illumination than in bulk materials. In recent years, many semiconductor nanostructures, such as In_2_Se_3_ nanobelt, ZnSe nanowire, CdS nanobelt, and ZnO nanowire [1-4], have been used to fabricate nanoscale photosensitive devices. However, most of the nanostructures are observed to have a low photocurrent response of <10^2^ and a slow response time of >1 s, which cannot meet the basic requirement for practical application in photosensitive devices. Therefore, researchers have been looking for new nanomaterials as candidates.

As one of the important III-V semiconductors, AlN nanostructures have been paid much attention in recent years. It is known that AlN nanostructures have the same high thermal conductivity (*K* ~ W/m·k), high melting point over 2,300°C, and large direct bandgap (6.28 eV) [5-8] as those of their bulk materials, which are suggested that they should be the promising fundamental blocks in building nanoscale optoelectronic devices. Although there have been many research groups focusing on the synthesis methods of AlN nanostructures, a few reports are devoted to investigate their photoconductive performances. There are a series of difficulties existing in the research of the photosensitive properties of AlN nanostructures, which can be described as the following: (1) AlN nanostructures with uniform morphology are hard to synthesize in large scale; (2) AlN nanostructures have poor conductivity of about 10^−8^ to 10^−6^ Ω^−1^·cm^−1^ in most reports [9-11], which is too low to be applied on optoelectronic nanodevices in general; and (3) AlN nanostructures have relatively lower lengths (<2 μm) in many experiments, which is too difficult to manipulate for nanodevices. To promote the rapid development of AlN nanomaterials in photosensitive applications, the researchers must solve these difficulties in advance.

Here, we report the synthesis and characterization of ultra-long AlN nanowire (UAN) arrays by chemical vapor deposition (CVD). The photoconductive behaviors of individual UAN under different excited lights are compared together. Moreover, their photoconductive mechanism is illustrated by a combination of two existing models.

## Methods

**Figure 1:**
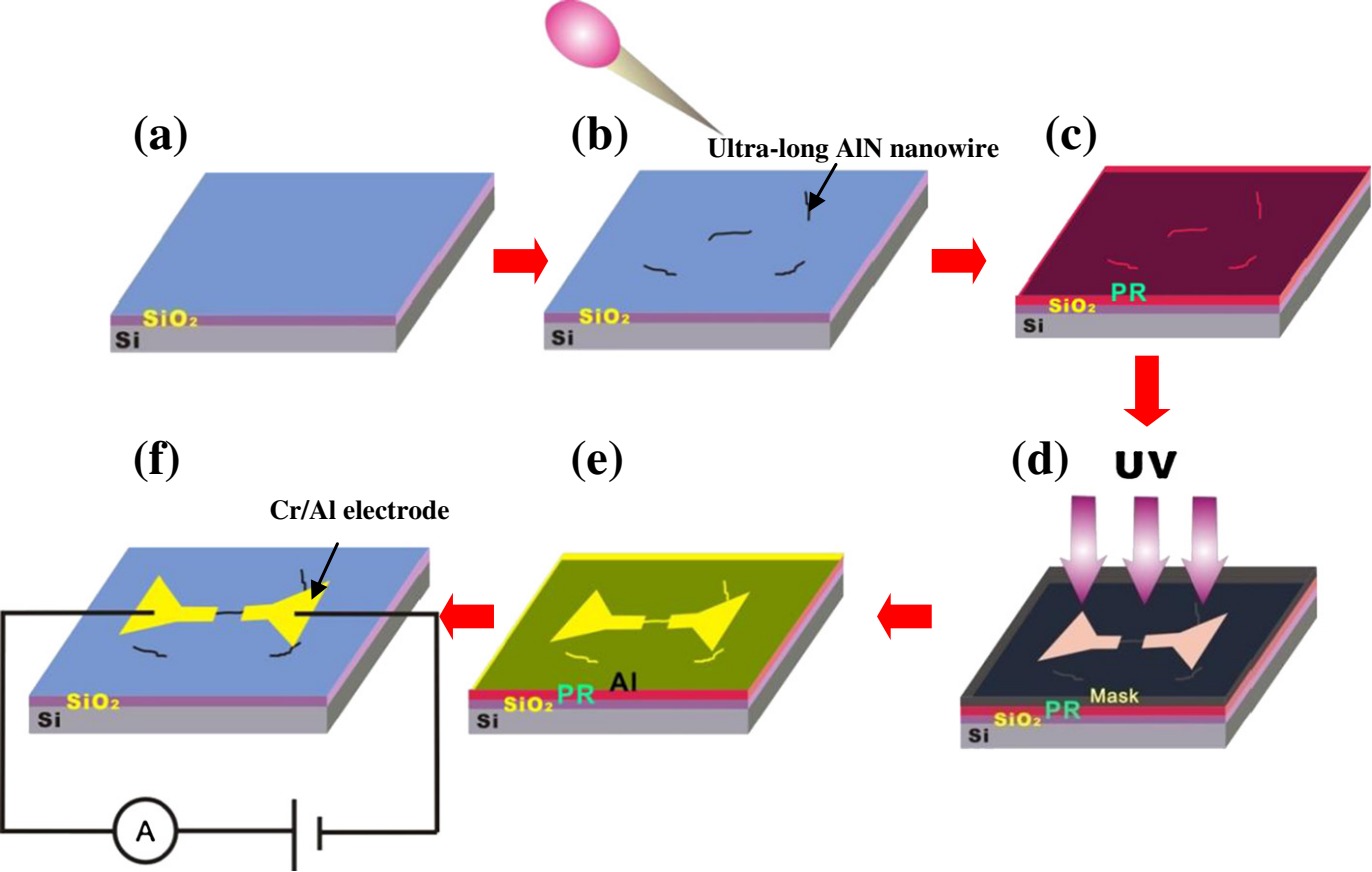
Schematic diagram of a series of ultraviolet lithography technique to fabricate individual UAN photoconductor.

Large area UAN arrays have been successfully synthesized on Si substrate using Al and Fe_2_O_3_ powders as source materials [12]. The growth system has been described in our recent reports [12-15], and the substrate is silicon wafer. Carrier gases consisted of N_2_ and NH_3_ with a flow rate ratio of 200 to 20 sccm, and they are introduced into the vacuum chamber when the temperature reaches approximately from 1,000°C to 1,200°C. Growth pressure was kept at 50 Torr in the reaction process. Detailed preparation procedure of individual UAN photoconductors is shown in Figure 1. Firstly, the silicon substrate is oxidized to form an insulated SiO_2_ layer with a thickness of about 300 nm in the air. Secondly, the as-prepared UANs (>50 μm) are spread over the substrate after they are deeply dispersed in ethanol solution by an ultrasonic wave cleaner. Thirdly, a layer of photoresist is used to cover the UANs by spin-coating technique. Fourthly, single UAN nanowires are selected to be treated by a series of simple ultraviolet photolithography technique. Fifthly, the Cr/Al electrodes are deposited on both ends of the individual UAN. Finally, the substrate is immerged into acetone solution to lift off the photoresist layer, and individual UAN is integrated into the single photoconductor by linking with the outside electric circuit.

Transmission electron microscopy (TEM) (Tecnai-20, Philips Tecnai, Amsterdam, The Netherlands) and X-ray diffraction (XRD) (RINT 2400, Rigaku Corporation, Tokyo, Japan) techniques were used to research on the crystalline structures of UANs. Their optical property measurements were performed on a HITACHI F-4500 type spectrophotometer (Hitachi High-Tech, Minato-ku, Tokyo, Japan) with a 325-nm He-Cd laser as the excitation source. In addition, the working performance of a single UAN under different excited lights was investigated in our self-built measurement system.

## Results and discussion

**Figure 2:**
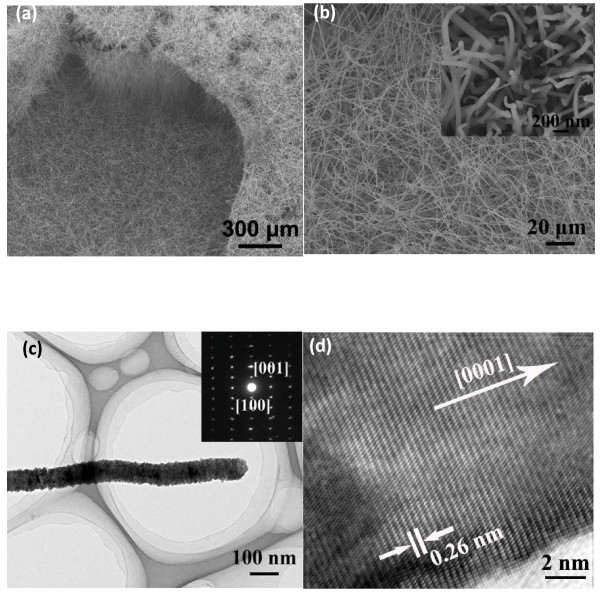
**SEM and TEM images UAN.** (**a**) Top-view and (**b**) side-view SEM images of UAN arrays. (**c**) Typical TEM and (**d**) HRTEM images of an individual UAN. The SAED pattern of the nanowire is in the inset of (c).

UAN arrays in high density were prepared on Si substrate by optimizing the reaction conditions, and the growth process has been depicted in our recent paper [12]. Figure 2a,b respectively gives the side-view and top-view images of UAN arrays. In Figure 2a, it is seen that the UANs stand on the silicon substrate with an angle of about 60° and that their growth density is very high. Moreover, the length of the AlN nanowire is ranging from 60 to 80 μm, which is very helpful for the photolithography process because it can be easily handled in the optical microscope. It is observed from Figure 2b that the UANs have uniform morphology with coarse surfaces. Their mean diameter is seen to be about 100 nm, and their aspect ratio reaches 700. Their similar morphology can ensure that single UAN photoconductors with uniform optoelectronic properties can be constructed. Their growth mechanism is attributed to the self-catalyzing vapor–liquid-solid mechanism, as described in [12].

**Figure 3:**
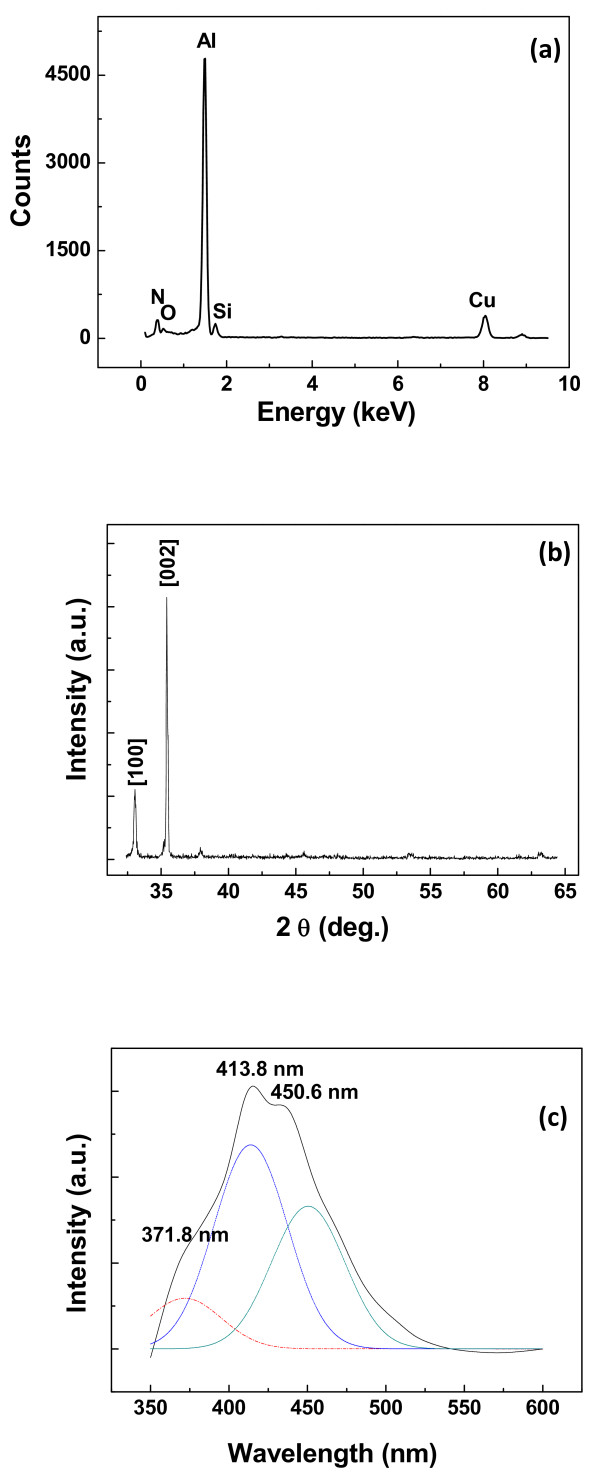
**EDX spectrum, XRD pattern, and PL spectrum of nanowires.** (**a**) Representative EDX spectrum of a single AlN nanowire. (**b**) The XRD pattern of the UANs. (**c**) Typical PL spectrum of UAN arrays.

The crystalline structure of UAN is investigated by TEM and high-resolution TEM (HRTEM) techniques. Typical TEM and HRTEM images of a UAN are respectively given in Figure 2c,d. The UAN is confirmed to have a coarse surface in Figure 2c, which is consistent with the SEM result in Figure 2b. Although a coarse structure is observed on the surface of the AlN nanowire, they are still single crystals from the clear selected area electron diffraction (SAED) pattern (inset). It is also seen that the diffraction spots have been lengthened to some extent, which suggests that some crystalline defects or other doping elements may exist in the lattice. Their atom-resolved TEM image (Figure 2d) is taken from the same nanowire in Figure 2c, and the distance between adjacent layers is about 0.26 nm along the growth axis. In addition, there perhaps exist a few defects or other doping elements in the lattice, which can be found in the left corner of the image. The representative EDX spectrum of a single AlN nanowire is provided in Figure 3a for the analysis of the element compositions of an individual AlN nanowire. The total content of element Al and N is found to be over 97%. It is also found that the content of element Si is about 1.2%, which may originate from Si doping of the substrate or quartz tube in the high-temperature growth process. The O content is about 0.8% which probably comes from the oxygen-related defects or the surface adsorbents, and element Cu should come from the TEM grid. By combining the SAED pattern and the HRTEM image, the UANs are determined to be single crystals with wurtzite structures, and they grow along the [0001] direction. To further confirm the chemical compositions and the crystallinity of the UAN arrays, XRD technique is applied on the sample. In Figure 3b, it can be found that the peaks are very sharp and that they are consistent with the data of the JCPDS (card no. 25–1133). Moreover, it is clearly seen that only two diffraction peaks are observed in this pattern and that the intensity of [100] peak is much lower than that of [002] peak. Thus, a conclusion can be drawn that UANs are single crystalline nanostructure arrays with good alignment.

Optical properties of UAN arrays are firstly investigated using a photoluminescence (PL) spectrophotometer before they are built into photoconductors, as shown in Figure 3c. Here, the excitation wavelength of 325 nm is applied on UAN arrays at room temperature, which is the minimum wavelength for the laser in this equipment. It can be seen that there exists a wide emission band, as shown in Figure 3b, which ranges from 350 to 525 nm. Using the Gaussian line fitting, the broad emission band can be divided into three peaks, which are respectively located at 371.8, 413.8, and 450.6 nm. It is known to us all that the basic equation of phonon energy can be written as follows:

(1) $$h\gamma =h\frac{c}{\lambda }=E,$$

where *E* is the photon energy, *h* is the Planck constant (6.63 × 10^−34^ J·s), *γ* is the frequency of the emission light, *c* is the light velocity (3×10^8^ m/s), and *λ* is the wavelength of the emission light. Hence, the peak at 371.8 nm corresponds to the energy level of 3.34 eV in the bandgap; the peak at 413.8 nm comes from the energy level of 3.01 eV, and the peak at 450.6 nm is from the energy level of 2.76 eV, based on this calculation method. According to the reports of other researchers [16-18], the weak ultraviolet light peak centered at 371.8 nm and the blue light emissions at 413. 8 nm should be attributed to the existence of oxygen impurities in the nanowire. In general, the oxygen atoms substitute for nitrogen sites in the lattice, and they usually form the oxygen point defects, $O_{N}^{+}$, nitrogen vacancies (*V*_N_), and $V_{\text{Al}}^{3-}+3O_{N}^{+}$ complexes [19-21]. Their corresponding energy levels should be located in the mid gap of AlN nanowires, which result in the formation of the two PL peaks. The oxygen contents are suggested to have two possible sources: one is that the oxygen atoms can be released from the quartz tube used in the high-temperature growth process, and the other possibility is that the oxygen atoms are absorbed on the surface of the nanowire after the sample is removed from the preparation vacuum chamber. In our experiment, the 450.6-nm peak is related to the N vacancy due to the nonstoichiometric composition in the reaction [18-21]. The broadening of the PL spectrum of the AlN nanowire ascribes to the oxygen-related defects and the surface adsorbents. Because the deficiency of oxygen or nitrogen leads to the formation of the deep donor or acceptor levels in the AlN bandgap, the photo-generated electrons and holes simultaneously occur in the conduction band and the valence band under illumination of a 325-nm light. The deep donor levels can provide the recombination centers for these carriers in the energy gap of AlN, and radiative luminescence will be found in the PL measurement, as observed in Figure 3c.

**Figure 4:**
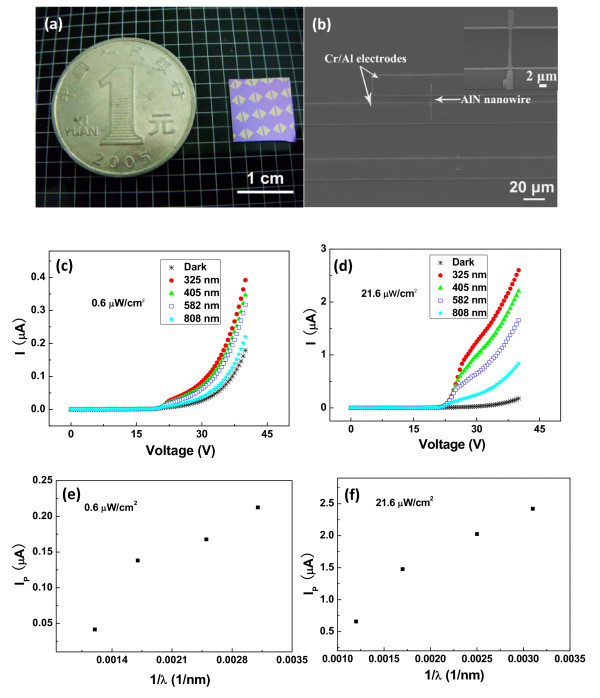
**Magnification images and*I*-*V*curves of single UAN and photo-generated current versus light wave number curve.** (**a**) Low magnification and (**b**) high magnification images of a single UAN photoconductor. (**c**, **d**) The *I*-*V* curves of a single UAN at an irradiance of 0.6 and 21.6 μW/cm^2^, respectively. The curve of (**e**) photo-generated current versus (**f**) the light wave number at an illumination irradiance of 0.6 and 21.6 μW/cm^2^, respectively. The applied voltage is 40 V.

**Figure 5:**
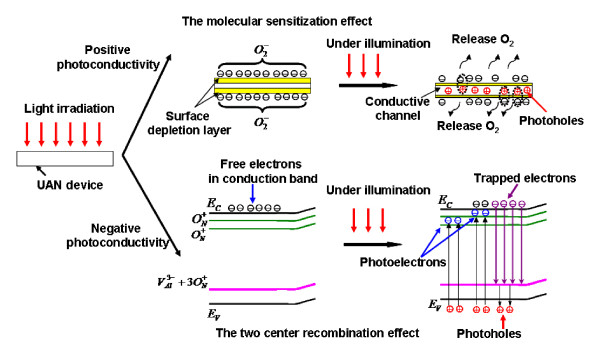
Illustration of the photosensitive mechanisms of single UANs.

The valuation of the room-temperature photoconductive behaviors of UANs is necessary for their future optoelectronic applications, so we carried out the experiments in our self-built measurement system. A pico-amperemeter (model 6487 Keithley Instruments Inc., Cleveland, OH, USA) with a controlled program is used to auto-record the experiment data and provides the applied voltage in measurements. Figure 4a is a photograph of single UAN photoconductors and a ¥1 RMB coin. As reported in [12], individual AlN nanowires in our experiments have a relatively higher electrical conductivity of about 2 to 4 × 10^−4^ Ω^−1^·cm^−1^ than those (approximately 10^−7^ to 10^−6^ Ω^−1^·cm^−1^) in other preparation methods [10]. It is seen that nine single nanowires are respectively integrated on a piece of Si substrate with a cover layer of SiO_2_. The size of the substrate is about 1×1 cm^2^, which is obviously smaller than that of the ¥1 RMB coin. A high-magnification SEM image of a UAN photoconductor is given in Figure 4b. It is seen that the width of the Cr/Al electrode is 20 μm and that the distance between adjacent electrodes is 10 μm. In the inset, it is also seen that only one nanowire existed between two adjacent electrodes, and the contact between the nanowire and the electrodes seem to be very tight. The *IV* curves of individual UAN under different wavelengths of light are given in Figure 4c, and the irradiance of the excited light is kept at 0.6 μW/cm^2^. For comparison, the *IV* curves of individual UAN under the excited light of an irradiance of 21.6 μW/cm^2^ are provided in Figure 4d. From these curves, we can see that the photo-generated current increases with the decrease of the wavelength of the stimulated light. To further compare their photoconductive performance, the curves of photo-generated current versus the light wave number at these two illumination irradiances are respectively shown in Figure 4e,f. The same tendency of the photocurrent with the light wave number can be obviously found, which suggests that the efficiency of photo-generated carriers is dependent on the wavelength of stimulated light rather than the illumination irradiance. The distinct positive photoconductive phenomena to these given excited lights may be explained by the combination of two mechanisms. The positive photoconductive response of individual UAN is attributed to the molecular sensitization model in some nanostructure systems [22-24]. The graphic illustration of photosensitive mechanism of single UAN is indicated in Figure 5. According to the molecular sensitization mechanism, oxygen molecules can be adsorbed on the surface of AlN nanowires during or after the growth process, and they form negative oxygen ions, $O_{2}^{-}$, by capturing some free electrons from AlN semiconductors. Free electrons are driven away from the surface of AlN nanowires by these adsorbed negative oxygen ions, and the surface of the nanowires becomes the electron depletion layer, which can be seen in the top image of Figure 5. As a result, the conductive channels of the electron in the AlN nanowire turn narrow, which makes the resistance of the nanowire larger than before. When the nanowire is under illumination, the photo-generated holes can move forward to the depletion layer and neutralize these $O_{2}^{-}$ to form oxygen gas at the function of a built-in field. Because of the release of the oxygen gas, the resistance of the nanowire will turn smaller with the increasing of the electron channel width, which leads to the positive photoconductive response, whereas the two-center recombination effect is used to express the negative photoconductive behaviors [24,25]. Based on the two-center recombination mechanism, the oxygen atoms or other elements firstly form a series of impurity levels in the energy gap of AlN, which conforms to our PL results (Figure 3b). The upper electron trapping levels ( $O_{N}^{+}$) and the acceptor levels (*V*_N_ and $V_{\text{Al}}^{3-}+3O_{N}^{+}$) should coexist in the bandgap of the nanowire. Under the illumination of the excited light, the photo-generated electrons can mirage to a high donor level ( $O_{N}^{+}$) close to the conduction band. At the same time, the photo-generated holes can recombine with the trapped electrons at a lower impurity level close to the valence band, as illustrated in the bottom image of Figure 5. The remained donor levels ( $O_{N}^{+}$) will capture the emission electrons from the conduction band, which gives rise to the negative photoconductive behaviors of the nanowire. As for the as-prepared UANs in our experiments, both of these two effects possibly take place in our nanowire system at the same time. However, the molecular sensitization effect will dominate over the electron emission process rather than the two-center recombination effect because single AlN nanowires perform positive photoconductive behaviors in our measurements. It can be explained as follows: one is that the concentration of the defects $O_{N}^{+}$, *V*_N_, and $V_{\text{Al}}^{3-}+3O_{N}^{+}$ in UANs should be very low at high growth temperature (>1,000°C) process; the other is that the content of the surface oxygen ions is high enough because UANs have a very large surface volume, which is in agreement with their HRTEM results.

Moreover, the device sensitivity or the photocurrent-to-dark current ratio (*I*_P_/*I*_D_) of a single UAN at a low irradiance of 21.6 μW/cm^2^ is almost 20 times bigger than the dark current at the same excited light of 325 nm, which is comparable to many other (In_2_Se_3_, ZnSe, ZnO, and AlN nanowires) nanostructures [1-3,24]. It is also found in Figure 4c,d that single UAN has a higher sensitivity (20) at an irradiance of 21.6 μW/cm^2^ than at an irradiance of 0.6 μW/cm^2^ (3). It can be interpreted that the number of the photo-generated electrons and holes will turn larger with the increase of the illumination irradiance, which enhances their photoconductivity. The nonlinear relationship between the photocurrent and the applied voltage is observed here, which may originate from the existence of the Schottky barrier in the measurement. As similar as our recent studies [12,14], the total resistance, *R*_Total_, in the measurement should consist of two kinds of resistances, which are the contact resistance (*R*_Contact_) between the nanowire and the Cr/Al electrodes and the intrinsic resistance (*R*_AlN_) of the AlN nanowire, and their relationship can be written as follows:

(2) $$R_{\text{Total}}=R_{\text{AlN}}+R_{\text{Contact}}\text{.}$$

The work function of the Cr electrode is about 4.6 eV, which is higher than that of the AlN (3.8 eV). Thus, the Schottky barrier should occur when they are built in nanometer devices, which induces that their *IV* curves exhibit nonlinear relationships. Based on the improved metal-insulator-vacuum (MIV) model in our recent work [14], the *IV* curves at high voltage can reflect the native conductive behaviors of the nanowire because the Schottky barrier at high applied voltage will be tunneled through and will decrease much lower than the intrinsic resistance of the nanowire. In our measurement, the photocurrent at high voltage is almost proportional to the applied voltage, which proves that the photosensitive process of individual UAN under illumination should obey the improved MIV model. In other words, the photosensitive performance to several wavelengths of light resulted from single AlN nanowire rather than the Schottky barrier.

**Figure 6:**
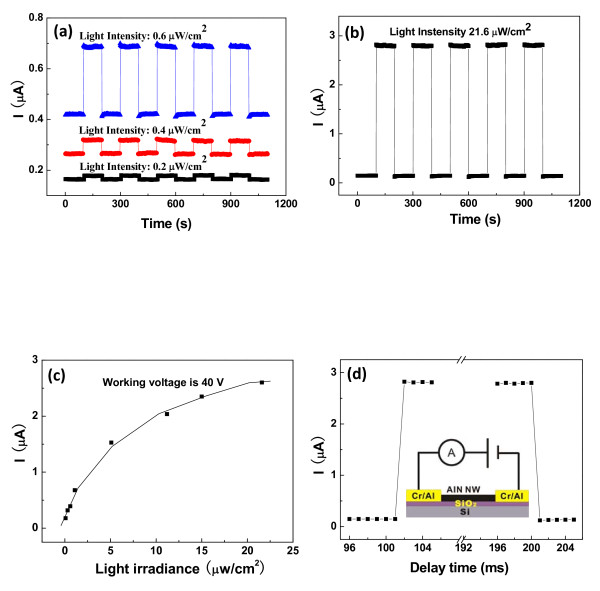
**Time response, different irradiance values, and response curve.** (**a**, **b**) The time response of a photocurrent at different irradiances of 0.2, 0.4, 0.6, and 21.6 μW/cm^2^ under 325 nm of UV light illumination over 5 cycles. (**c**) Photocurrent under a 325-nm illumination with different irradiances (0.086, 0.3, 0.6, 1.1, 5.1, 11.2, 15, and 21.6 μW/cm^2^). The working voltage is 40 V. (**d**) The fast turn-on and turn-off time response curve of a single UAN at ranges of 96 to 105 and 196 to 201 ms. The inset gives the representative measurement electric circuit of an individual UAN.

Although single UANs are more sensitive to 325 nm UV light than other wavelengths of light, the time response of a photocurrent at different light intensity of 0.2, 0.4, 0.6, and 21.6 μW/cm^2^ is specially provided under this wavelength of light over 5 cycles in Figure 6a,b. Resembling to their photosensitive behaviors in Figure 3c,d, individual UANs at higher illumination irradiance has a better performance than at low irradiance. In addition, single UANs have very good working stability over 5 cycles whether they are under what irradiance. Figure 6c gives the photocurrent as a function of different luminance irradiances (0.086, 0.3, 0.6, 1.1, 5.1, 11.2, 15, and 21.6 μW/cm^2^) of the excited light. The applied voltage is kept as 40 V, and the wavelength of the light is chosen to be 325 nm in experiments. The photocurrent is observed to have a nonlinear relationship with the luminance irradiance. It can be comprehended that there is a simple power law existing between the photocurrent and the luminance irradiance, which is expressed as [26] follows:

(3) $$I_{P}\propto P^{0.94},$$

where *P* is the luminance irradiance of the stimulated light, and *I*_P_ is the photocurrent. According to this equation, the photocurrent should nonlinearly increase with the luminance irradiance, which is consistent with our experimental results. The fast turn-on and turn-off time response curve of individual UANs at ranges of 96 to 105 and 196 to 201 ms is indicated in Figure 6d to test the response speed of the UAN photoconductor. From Figure 6d, it is seen that a single UAN has a very fast response time of about 1 ms, which has reached the measurement limit of our present system. The inset gives the measurement electric circuit. It can be expected that if the switch of the system can be auto-controlled by a faster program, then the turn-on or turn-off time of the single UAN will be decreased to a much lower level of microseconds. To better value the photosensitive performance of single UANs, we summarized the photosensitive behaviors of different nanostructures in TableÂ 1. By analyzing this table, it is found that the current fluctuation on an applied voltage of 40 V is smaller than 2% in a period of two continuous working hours, which is better than many nanostructures [1-4]. The current response *R*_λ_ is an important device parameter to testify the performance of the photoconductor, which corresponds to their sensitivity to light. It can be calculated as the following [1,27,28]:

(4) $$R_{\lambda }=I_{P}/P_{\lambda }S,$$

where *I*_P_ is the photocurrent, *P*_*λ*_ is the light intensity, and *S* is the effective illuminated area of the nanowire at the irradiation. Based on this equation, the detector current response *R*_*λ*_ of a single UAN is over 2.7×10^6^, which is much bigger than many nanostructures. Although their operating voltage seems to be higher in some content, effective element doping should decrease their operating voltage to several voltages by improving their conductivity. Considering their better photoconductive performance, individual UANs are suggested to have a very promising future in photosensitive applications if their conductivity can be controllably modulated.

**Table 1:** Comparison of the room-temperature photoconductive performance of single UANs with other nanostructures

	**Photosensitive properties**					
**Nanostructure**	**Operation voltage (V)**	**The ratio of photocurrent to dark current (*I***_**P**_**/*I***_**D**_**)**	**Detector current responsivity (*R***_***λ***_**) (A/W)**	**Response time**	**Device stability fluctuation**	**Reference**
In_2_Se_3_ nanobelt	3	5	89	<0.3 s	<10%	[22]
ZnSe nanowire	30	Approximately 10	0.12	<0.3 s	<8%	[23]
CdS nanobelt	1	Approximately 10	7.3×10^4^	Approximately 20 μs	<3%	[24]
ZnO nanowire	2	8	Not given	Approximately 50 s	<20%	[25]
AlN nanowire	10	4.5	Not given	Approximately 1 s	<5%	[20]
UAN	40	20	2.7×10^6^	Approximately 1 ms	<2%	In this research

## Conclusions

UANs with length over tens of micrometers have been successfully fabricated in a large area by CVD method, and they are integrated into nanometer photoconductors by a simple ultraviolet photolithography technique. Individual UANs are found to have good photoconductive behaviors under some given excited lights, such as a faster response time of about 1 ms, higher photosensitivity (20), and more stable working performance. Their photosensitive mechanism is attributed to the combination of the molecular sensitization effect and the two-center recombination effect. UANs should be a very good candidate in photosensitive applications if their optical properties can be further controlled by element doping.

## Competing interests

The authors declare that they have no competing interests.

## Authors’ contributions

FL and LFL carried out the growth of the AlN nanowires. TYG, HBG, and XSM participated in the measurement of the physical properties of individual AlN nanowires. JC, SZD, and NSX contributed in the drafting and revision of the manuscript. All authors read and approved the final manuscript.
